# Research on the Improvement Path of Human-AI Collaborative Consultation Effectiveness From the Perspective of Information Ecology: Configurational Analysis

**DOI:** 10.2196/90611

**Published:** 2026-07-15

**Authors:** Dandan Wang, Mengyi Zhang, Bingjun Guo

**Affiliations:** 1 Business School, Henan University of Science and Technology, Business School, Henan University of Science and Technology, No. 263 Kaiyuan Avenue, Luolong District, Luoyang, Henan, 471023, China, 86 1-503-698-1959; 2 School of Computer Science and Information Engineering, Anyang Institute of Technology, Anyang, Henan, China

**Keywords:** online health community, human-AI collaborative consultation effectiveness, patient satisfaction, information ecosystem, configuration path

## Abstract

**Background:**

Enhancing the effectiveness of human-AI collaborative consultation in online health communities (OHCs) constitutes a core requirement for optimizing the allocation of medical resources and promoting the sustainable development of medical services. Nevertheless, the pathways to improving such effectiveness remain insufficiently understood.

**Objective:**

This study aimed to conduct an in-depth exploration of the multiple factors influencing the effectiveness of human-AI collaborative consultation in OHCs and to assess the causal relationships among these factors, thereby providing a theoretical foundation and practical guidance for advancing the clinical application of human-AI collaboration.

**Methods:**

Grounded in the information ecology theory, we constructed an analytical framework encompassing four dimensions: information human, information, environment, and technology. We collected 296 valid questionnaire responses and used fuzzy set qualitative comparative analysis to systematically investigate the configurational mechanisms through which these four types of factors jointly influence consultation effectiveness.

**Results:**

The findings are as follows: (1) no single factor constitutes a sufficient condition for high consultation effectiveness (consistency <0.9); rather, such effectiveness emerges from the synergistic interplay of multiple configurations involving technology, information, human information, and environment; (2) five distinct configurational pathways lead to high consultation effectiveness, demonstrating clear equifinality; (3) system responsiveness, information usefulness, perceived service empathy, perceived service accuracy, and perceived service effectiveness are all core or important conditions across these pathways; and (4) substitutability exists among antecedent conditions—specifically, perceived uncertainty and social norms, as well as operational convenience and platform ethical norms, can substitute for one another in different configurations to enhance patient satisfaction jointly.

**Conclusions:**

This study reveals multiple pathways to achieving high effectiveness in human-AI collaborative consultation within OHCs. It not only offers a novel theoretical perspective for understanding the complex mechanisms of human-AI collaboration in medical contexts, but also provides significant practical implications for the design optimization of digital health platforms and related policy formulation.

## Introduction

### Background

In recent years, with the rapid development of information technology and the advancement of the “Healthy China” strategy, smart health care has garnered extensive attention from national policies and social capital. The “Healthy China 2030” Plan Outline clearly states that health should be granted strategic priority in development, and that information technology should be fully used to promote the development of smart health care and enhance the accessibility and quality of medical services [1]. Against this backdrop, the number and scale of online health communities (OHCs), such as Haodafu, Chunyu Yisheng, and Dingxiangyuan, have grown significantly, demonstrating substantial development potential [2]. Nevertheless, although technological innovations represented by “Ping An Yibotong” and “Jingyi Qianxun” are driving the transformation of consultation services toward intelligence and efficiency, the service quality and effectiveness of these platforms in actual application remain in need of improvement. Currently, patients’ satisfaction and willingness to use intelligent consultation services are generally low, and trust in human-AI interaction is markedly lacking [3]. Therefore, effectively enhancing the effectiveness of human-AI collaborative consultation is not only related to the optimization of service processes and the resolution of user trust predicaments, but also the key to fully unleashing the potential of intelligent diagnosis, which is of great significance for promoting the sustainable development of smart health care.

Existing research has primarily approached the issue from single perspectives, including perceived value [4], doctor-patient interaction [5], perceived risk and service quality [6], the success of information systems and social support [7], doctor-patient trust [8], and information ecology [9]. These studies have explored the influence of factors such as perceived value, perceived risk, service quality, system quality, information quality, social influence, platform norms, and laws and regulations on patient satisfaction (PS) in OHCs. However, most existing studies focus on the independent effects of individual factors, while paying relatively less attention to the collaborative effects among information human, information, environment, and technology, as well as the dynamic adaptation relationships among various elements within the information ecosystem. Moreover, systematic research on the efficacy of the core model of human-AI collaborative consultation remains rather limited, resulting in a lack of systematic theoretical guidance for OHCs when optimizing their services.

In view of this, this study takes PS as the measurement index of the effectiveness of human-AI collaborative consultation, constructs a multidimensional analysis framework from the perspective of information ecology, and uses the fuzzy set qualitative comparative analysis (fsQCA) method to explore the configuration paths of information human, information, technology, and environmental elements. Specifically, this study addresses three key issues: (1) What elements of the information ecosystem interact to exert differential impacts on the effectiveness of human-AI collaborative consultation in OHCs? (2) What condition configurations exist to drive the improvement of human-AI collaborative consultation effectiveness in a “multiple concurrent” manner? (3) Which antecedent conditions are more crucial for enhancing the effectiveness of human-AI collaborative consultation?

The theoretical contributions and innovations of this study are mainly reflected in three aspects: first, it breaks through the limitation of a single perspective and constructs a 4D integrated analysis framework based on the information ecology theory, systematically analyzing the mechanism of the effectiveness of human-AI collaborative consultation, enriching the related research system; second, it introduces the fsQCA configuration thinking, breaking away from the limitations of linear analysis, exploring the concurrent driving paths of multiple elements, and revealing the nonlinear mechanism of efficiency improvement; and third, it closely focuses on the actual scenarios of human-AI collaboration, bridging the gap between theory and practice, providing a practical theoretical basis for the optimization of OHC services. Furthermore, human-AI collaboration is a common trend in the global development of smart health care. Although this study is rooted in the specific context of Chinese OHCs, the core issues faced by human-AI collaborative consultation have certain commonalities in different medical systems and cultural backgrounds. Therefore, the analysis framework and the revealed configurational path constructed in this study are expected to provide transferable theoretical references and practical inspirations for digital health platforms facing similar trust dilemmas and the need for performance improvement worldwide.

### Prior Work

#### Theoretical Basis

The information ecology theory was proposed by Horton [10] in the 1980s, emphasizing the harmonious symbiotic relationship among “information human, information, environment, and technology” in the information system, and focusing on the interdependence, interaction, and dynamic evolution process among various elements of the system. Among them, the information person is the main body of the system, leading the initiation and interaction of information activities. Information exists as an object, carrying knowledge, data, and meaning. Technology provides tool support for the processing and transmission of information. The environment constitutes the field where information human, information, and technology interact, shaping the patterns and boundaries of information behavior [11]. Subsequently, Davenport [12], and Nardi and O’Day [13], among others, further developed this theory, defining it as “the relationship between people and organizations in the information environment,” viewing “the information ecosystem” as “an organic system of people, practices, values and technologies in a specific environment,” and pointing out that the information ecology refers to a dynamic equilibrium state achieved through the transmission and feedback activities of information human and the information environment within a specific information space, using information technology as a means [14]. This theory emphasizes the application of ecological methods to study the interaction mechanisms among system factors [15], with the goal of achieving “information ecological balance” [16]. In conclusion, the information ecosystem is an open and organic whole with human beings as the core element, and there exists an information flow relationship among these elements.

The existing technology acceptance model focuses on linear cognitive drive, while the SERVQUAL (service quality theory) concentrates on the gap between expectations and perceptions. Both of these approaches are unable to explain the nonlinear interaction and multifactor synergy in human-AI collaborative medical consultation. The social technology system theory, although focusing on the interaction between social and technological subsystems, lacks a sufficient depiction of “information” as an independent element and external environmental norms. The institutional perspective mainly emphasizes the macrolevel and is difficult to integrate with the microperception variables of individual patients. The information ecology theory, on the other hand, provides a more macro and dynamic systemic lens, emphasizing the interdependence, coevolution, and adaptive configuration among the four elements of “information human, information, technology, and environment.” The human-AI collaborative medical consultation process in OHCs is essentially a process of information flow and reconfiguration, constituting a typical information ecosystem [17]. In this system, patients act as information human to initiate interactions, diagnostic information flows as the core object, intelligent systems and platforms provide technical support, and regulations and ethical norms form the environmental field. All four elements are present. The consultation process presents a continuous information cycle: patients describe symptoms, AI assists in triage, doctors provide diagnosis feedback, patients generate efficacy feedback after execution, and this triggers new information needs, forming a multidirectional interaction network. These elements are deeply coupled and interact with each other, jointly determining the effectiveness of the consultation service.

Based on this theoretical perspective, the core variable system constructed in this study is not a simple superposition of existing theories, but a systematic logical reconstruction: the classic constructs of technology acceptance and service quality are respectively anchored in the “information technology” and “information person” dimensions, and the attribute of information is independently classified as the “information” dimension and incorporated into the ecological regulatory field of “environment.” This design transcends the explanatory boundaries of traditional linear models and, by examining the collaborative configuration of “person-information-technology-environment,” reveals the complex causal chain behind the effectiveness of human-AI collaborative medical consultation, providing a more systematic theoretical basis for optimizing the digital health service ecosystem.

#### Research on Human-AI Collaborative Consultation in OHCs

As an important tool for integrating multiple resources and enhancing the effectiveness of medical services, OHCs have transcended traditional information exchange spaces and become platforms that support users in sharing experiences, consulting experts, and promoting knowledge cocreation [18-20]. With the help of Web 2.0 technology, these communities provide services such as health and medical information, online consultation, and knowledge question and answer, building a virtual communication space for patients, doctors, and health enthusiasts [21,22]. Human-AI collaboration aims to establish a deeply integrated human-AI interaction model by integrating the advanced cognitive abilities of humans with the technical advantages of machines, achieving complementary strengths and efficient collaboration [23,24]. This system framework emphasizes handing over the final decision-making power to personnel with professional knowledge and experience, in order to overcome model uncertainties and ensure the reliability and stability of decisions [25]. In medical human-AI collaboration, AI systems actively identify user preferences, analyze vast medical literature and clinical cases, and accordingly provide diagnosis and treatment suggestions to doctors. Doctors then exercise their subjective initiative to evaluate, modify, or appropriately adopt these suggestions [26,27]. Through this dynamic coordination and efficient cooperation, machines focus on handling structured tasks, while humans concentrate on higher-level judgments and innovations. Both parties exchange information through specific interaction methods to jointly achieve work goals, thereby effectively improving service quality and work efficiency, and achieving the core goal of reducing burden and increasing efficiency [28-30]. In conclusion, the existing literature mostly focuses on the technical aspects of human-AI collaboration mechanisms, but pays insufficient attention to the specific context of OHCs. Therefore, this paper defines the human-AI collaborative consultation in OHCs as a process in which patients interact with AI systems and professional doctors on OHC platforms to obtain medical services such as health consultation and disease diagnosis. In this mode, patients input information through the platform. The AI system uses big data and algorithms to initially analyze and screen the symptoms, medical history, and other information input by the patients, and then provides auxiliary diagnostic suggestions for doctors. Doctors make a comprehensive judgment based on their professional knowledge and provide the final diagnosis and treatment plan.

#### Research on Influencing Factors of Human-AI Collaborative Consultation

Scholars have comprehensively used various qualitative and quantitative methods to systematically explore the influencing factors of PS in the human-AI collaborative consultation model, revealing its multidimensional and interactive complexity. In terms of quantitative research, relevant results rely on multiple theoretical models to clarify the interrelationships of key variables. The structural equation model reveals that functional value, social value in perceived value, as well as interactivity, perceived quality, etc, have a positive effect on satisfaction [4,31], while information accuracy (IA) and privacy security can indirectly affect satisfaction by influencing perceived benefits and costs [32]; Zhai et al [33] further subdivided the quality dimensions and verified that personnel quality, procedural quality, technical quality, and cost perception have differentiated effects on the satisfaction of remote consultation patients. Based on the unified theory of acceptance and use of technology model, the research focused on highlighting the core values of performance expectations, effort expectations, and social influence [34,35]. Meanwhile, related studies such as the perception risk model, the service quality model, and the trust theory have also confirmed the crucial restrictive and driving roles of perceived risk and doctor-patient trust in the consultation service [6,8]. Furthermore, studies from an information ecosystem perspective have found that environmental elements such as community interaction, atmosphere, and platform governance norms can effectively boost patients’ willingness to pay for consultation [22,36,37]. In OHC consultations, doctor-patient interaction and technical factors are particularly crucial. Wu et al [5] based on the dual-factor theory confirmed that the information support and emotional support provided by doctors are indispensable, and Deng and Hong [8] further emphasized the impact of website credibility. In qualitative and text analysis methods, different research methods have also formed complementary conclusions. Grounded theory related research has extracted multiple factors such as environment, platform, and physician services, which jointly affect user evaluation [38,39]; latent Dirichlet allocation model and Kano model further confirm the core roles of information quality, system quality, service quality, and privacy protection [9,21,40-42]; text mining analysis takes a communication perspective and confirms that the amount and comprehensibility of doctors’ responses significantly enhance satisfaction [43]. Meta-analysis–related results also verify the stable influence of the structural quality, process quality, and outcome quality of remote medical services [44].

In summary, although the existing studies have approached from multiple theoretical perspectives and widely identified various influencing factors, their common limitation lies in focusing on the independent net effect of a single or a few factors. This paradigm often presupposes linear relationships and independence between variables, resulting in a lack of a systematic integration framework that considers the four elements of information, information human, technology, and environment as an organic whole, and failing to deeply explore how multiple factors interact and collaborate dynamically in the specific scenario of “human-AI collaboration” to jointly affect consultation efficacy. It is difficult to reveal the possible multiple concurrent configuration paths for achieving high satisfaction, limiting the precision and situational adaptability of optimization strategies.

In view of this, this study introduces the perspective of information ecology and constructs an integrated analysis framework covering four dimensions: information human, technology, information, and environment. Focus on the analysis of the interactive relationship and collaborative mechanism of the four core elements during the consultation process; by applying fsQCA to explore the multiple concurrent condition combinations and equivalence realization paths that affect the effectiveness of human-AI collaborative consultation, we aim to break through the limitations of single-factor linear analysis and provide systematic theoretical support and practical guidance for precisely optimizing the service effectiveness of OHCs and promoting their sustainable development.

### Research Framework

#### Overview

This paper first conducts a literature analysis to sort out the relevant research, identifying various potential influencing factors under the four core dimensions of information human, information, technology, and environment. After expert consultation for revision and verification through practical cases, 13 specific influencing factors were ultimately determined, as shown in Table 1. Based on the essence of the information ecology theory and the scenario characteristics of human-AI collaborative consultation, it can be seen that the 4D elements do not exist independently but interact through complex relationships to jointly affect PS and the efficiency of the consultation service. Therefore, this paper will focus on the above four core dimensions to deeply analyze the connotations and mechanisms of action of each specific influencing factor, and construct a systematic theoretical model to reveal the formation logic and influence path of PS in the OHC’s human-AI collaborative consultation model.

**Table 1.** Key influencing factors of human-AI collaborative consultation in online health communities.

Factor categories and core dimensions	Brief connotation
Information factors	
Information accuracy	The accuracy of information obtained by patients during human-AI collaborative consultation.
Information usefulness	The usefulness of information or diagnostic results acquired by patients in the process of human-AI collaborative consultation.
Information understandability	The information provided by the human-AI collaborative consultation system is expressed in a clear and easy-to-understand manner, which is easy for patients to comprehend.
Information human factors	
Perceived uncertainty	The potential negative consequences that patients perceive they may face when using the human-AI collaborative consultation system.
Perceived service accuracy	Patients’ subjective judgment on the degree of alignment between medical services during consultation and their own health needs.
Perceived service effectiveness	Patients’ subjective perception of the speed and effectiveness of the services provided by the human-AI collaborative consultation system to meet their needs.
Perceived service empathy	The emotional understanding, care, and personalized attention provided to patients by the human-AI collaborative consultation system.
Technical factors	
System responsiveness	Patients’ subjective perception of the response speed, timeliness, and clarity of feedback of the human-AI collaborative consultation system.
System stability	Patients’ experience regarding the operational reliability, error rate, and compatibility of the human-AI collaborative consultation system.
Operational convenience	Patients’ feelings about the interface interaction logic, learning cost, and operational effectiveness of the human-AI collaborative consultation system.
Environmental factors	
Legal and regulatory protection	Patients’ evaluation of the performance of government departments’ responsibilities in leading, safeguarding, supervising, and managing the medical industry.
Platform ethical norms	The code of conduct formulated by the platform to ensure that consultations conform to ethics and norms.
Social rules	The degree of influence exerted by the words and deeds of people around patients and media publicity on patients’ use of the human-AI collaborative consultation system.

#### Information Human Factors

The information human factor reflects the subjective cognition and emotional response of patients in human-AI collaborative consultation, directly influencing their behavioral decision-making and consultation effectiveness. This study clearly defines the “information human” as the core entity that engages in information interaction, generates information needs, and perceives the consultation services in the human-AI collaborative consultation within OHCs. This entity is the patient who participates in the consultation and is also a user of this type of health service. To maintain the rigor and readability of the text, the following terminology guidelines will be followed: when presenting the theoretical model, “information human” will be used; while “user” or “patient” will only be used when referring to the research participants or the actors, to avoid confusion with the aforementioned core constructs. Patients’ concerns about potential risks such as medical quality, privacy protection, misdiagnosis risk, and waste of time or financial costs constitute perceived uncertainty (PU), significantly reducing trust and willingness to use. Perceived service effectiveness (PSEF), perceived service accuracy (PSA), and perceived service empathy (PSE) represent patients’ value judgments on core dimensions of system service quality, such as response speed, result reliability, and personalized care, and actively shape patients’ satisfaction and willingness to participate [31,45,46].

PU arises from concerns regarding the incompleteness of regulations and the immaturity of technologies associated with online consultation. It manifests as the perception of risks such as misdiagnosis, privacy leakage, and waste of time or money [35,47]. PSEF refers to a patient’s perception of the system’s ability to provide the required services quickly and effectively. PSA is the patient’s subjective judgment of the degree of alignment between the consultation service’s output and the patient’s own health needs. PSE refers to a system that delivers personalized and targeted services and care based on the patient’s medical condition and personal circumstances, thereby making the patient feel understood and valued. Collectively, these perceptions constitute the key psychological mechanisms underlying patients’ behavioral decisions, profoundly reflecting the subjectivity and initiative of the information human and its core driving role in human-AI collaborative interaction.

#### Information Factors

In online health consultation services, users’ adoption of inappropriate information may lead to serious consequences. Therefore, providing high-quality and highly persuasive information is of vital importance. Information quality is the key variable affecting user satisfaction [7] and encompasses three dimensions: information usefulness (IUS), IA, and information understandability (IUD). Together, these three dimensions form the basis for users to evaluate the value of information, and by influencing patients’ satisfaction, trust, and behavioral decisions, they shape the effectiveness of information adoption, usage, and dissemination, thereby sustaining the service quality and the overall viability of the information ecosystem in online human-AI collaborative consultation.

IUS refers to the perceived utility of the information or diagnostic results obtained by patients during the human-AI collaborative consultation process. IUS meets the core demand goals of users, which is the direct driving force for their continuous use and satisfaction. When patients truly experience the alleviation of health problems through human-AI collaborative consultation, such as reduced symptoms and decreased anxiety, the perceived usefulness of the information will significantly enhance their satisfaction and positively influence their willingness to share and continuously use the information [9,14,32,48].

IA refers to the degree of precision and reliability of the consultation information in aspects such as disease description, diagnostic conclusion, and prescription recommendation [49]. IA is the foundation for building trust and ensuring safety and reliability. Its mechanism of action is as follows: (1) accuracy of disease description: ensuring that the system precisely captures and structurally presents the details of symptoms, laying the initial trust of being understood. (2) Accuracy of disease diagnosis: relying on the deep collaboration between AI medical knowledge and doctors’ experience, a diagnosis that conforms to clinical guidelines and takes into account individual differences is produced, which triggers patients’ professional identity and strengthens their treatment expectations. (3) Prescription accuracy: ensuring the safety and effectiveness of the treatment plan through database verification and personalized adjustment, thereby enhancing patients’ confidence in the certainty of the diagnosis and treatment behavior. (4) Feasibility of suggestions: transforming professional information into action guidelines that fit patients’ life scenarios, thereby increasing their willingness to engage in health management. IA is an essential attribute for enhancing PS [21]. Higher IA directly strengthens patients’ trust in the system, confidence in treatment, and perception of service value, thereby significantly improving satisfaction.

IUD refers to the degree to which the content of the consultation information is expressed clearly and understandably [31]. IUD reduces users’ cognitive load and promotes the effective absorption of information. In the context of human-AI collaboration, if information presentation endures problems such as an accumulation of technical jargon, unclear logic, or obscure expression, it directly undermines users’ initial evaluation of service quality and leads to a decline in satisfaction. Conversely, the use of plain language, visualization tools, and similar strategies can effectively reduce the cost of understanding, enhance information acceptance, and improve PS [31].

#### Environmental Factors

The healthy development of online health human-AI collaborative consultation in health contexts relies on support from a stable multilevel environment. Legal and regulatory protection (LRP), platform ethical norms (PEN), and social rules (SR) interact synergistically, respectively, constructing the fundamental environment for system operation from the perspectives of rigid institutional constraints, organizational self-discipline, and sociocultural norms. These environmental factors, by shaping patients’ sense of institutional trust, organizational security, and behavioral predictability, reduce their perceived risks and jointly regulate and maintain the compliance, sustainability, and user satisfaction of the system.

LRP refers to patients’ evaluation of the fulfillment of leadership, guarantee, supervision, and management responsibilities by government departments in the medical industry. A comprehensive legal and regulatory system for internet health care, along with supporting mechanisms such as online medical dispute resolution and insurance coverage, reflects the state’s institutional commitment and governance capacity in this emerging field. Patients’ positive perception of policy effectiveness can effectively enhance their trust in the system [50], reduce concerns about systemic risks, and positively influence their evaluation of service satisfaction [51].

The PEN refers to patients’ evaluation of the system for protecting patient information security as formulated by the OHC platform. Ethical norms cover privacy protection, data security, and maintenance of user trust. The platform needs to make patients fully aware of its commitment and ability to protect personal information security through clear and transparent policy communication—such as minimizing data collection, implementing strict authorization mechanisms, and prohibiting data abuse [52]. High trust in the platform’s ethical norms can significantly alleviate patients’ perception of privacy leakage and data abuse risks, enhance their sense of organizational security, and serve as a key organizational commitment to maintaining user trust and improving satisfaction [53,54].

SRs refer to the extent to which the words and deeds of the people around the patient, as well as the influence of media publicity, affect the patient’s use of the human-AI collaborative consultation system. The pathways through which social norms influence patients are as follows: first, interpersonal influence and imitation—positive usage experiences and recommendations from relatives, friends, or similar groups can enhance patients’ confidence in using the new technologies and their expected efficacy [55,56]; second, media shaping and public opinion—positive media reports and a supportive social atmosphere created by public opinion reduce social resistance and cognitive uncertainty regarding the adoption of new technologies. Positive signals from SRs positively influence patients’ adoption willingness and satisfaction evaluations by reducing their social cognitive costs and perceived behavioral control deficits, while strengthening their value recognition of the services [51].

#### Technical Factors

System quality constitutes the core technical support for the implementation of human-AI collaborative consultation services in OHCs [57]. Based on the DeLone and McLean information systems success model, system quality is defined as a comprehensive assessment of system stability (SYS), system responsiveness (SYR), and operational convenience (OPC). These dimensions jointly form the technical framework of human-AI collaborative consultation. By optimizing system effectiveness, reducing usage barriers, and ensuring service reliability, they empower the efficient collaboration of other elements in the information ecosystem, ultimately driving the healthy development of PS and the service ecosystem [58-60].

SYR is defined as the subjective perception of patients regarding the feedback speed, timeliness, and clarity of the human-AI collaborative consultation system. SYR serves as the effectiveness hub of technical support, optimizing the timeliness of information transmission. High responsiveness not only enhances patients’ communication effectiveness but also increases their real-time satisfaction with the service experience. SYS refers to the patient’s experience of the reliability, error rate, and compatibility of the human-AI collaborative consultation system’s operation. SYS provides the fundamental carrying capacity of the technical environment, ensuring the continuous and reliable operation of services. Stable system operation can effectively reduce patients’ concerns about technical malfunctions and enhance their trust in the service and willingness to use it. The ease of operation refers to patients’ perception of the interface interaction logic, learning difficulty, and operation effectiveness of the human-AI collaborative consultation system. OPC is the user interface hub of technology landing, which directly affects the user experience and the effectiveness of patients. Convenient operation design can significantly reduce the learning cost of patients and enhance their acceptance and satisfaction with the system [61]. During the human-AI collaborative consultation process in OHC, high responsiveness ensures smooth communication, high stability guarantees continuous reliability, and high convenience enhances usage effectiveness. When patients perceive that the system offers fast response speed, operates stably without faults, and features a friendly interface, their satisfaction with the human-AI collaborative consultation service will be significantly enhanced.

**Figure 1.**
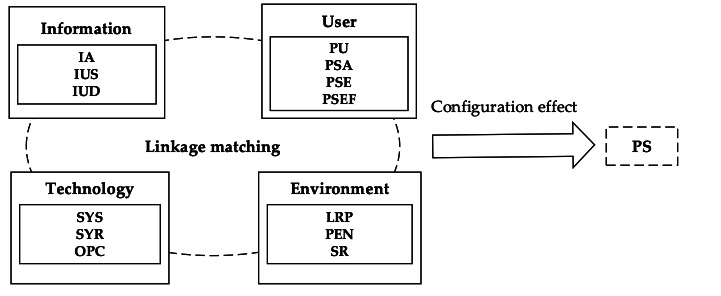
Research framework. IA: information accuracy; IUD: information understandability; IUS: information usefulness; LRP: legal and regulatory protection; OPC: operational convenience; PEN: platform ethical norm; PS: patient satisfaction; PSA: perceived service accuracy; PSE: perceived service empathy; PSEF: perceived service effectiveness; PU: perceived uncertainty; SR: social rule; SYR: system responsiveness; SYS: system stability

Based on the theory of information ecology and integrating the characteristics of human-AI collaborative consultation in OHCs as well as related research, this paper establishes a theoretical model of the influencing factors of PS under human-AI collaborative consultation in OHCs, as shown in Figure 1.

## Methods

### Method Selection

We used fsQCA to explore the complex causal mechanisms influencing the effectiveness of human-AI collaborative consultation in OHCs, for the following four reasons. First, this approach reveals nonlinear relationships between various antecedent conditions and consultation effectiveness, focusing on combinations of factors rather than the isolated effect of any single variable in isolation. Second, given our research question—“Which combinations of factors can lead to high consultation effectiveness?”—fsQCA enables the identification of multiple equifinal pathways that achieve the same outcome, thereby reflecting the principle of equifinality. Third, compared to alternative qualitative comparative analysis approaches, fsQCA is more suitable for handling continuous or ordinal variables, such as the Likert-scale items used in this study. Fourth, fsQCA accommodates a wide range of sample sizes, from very small datasets (fewer than 50 cases) to large-scale samples (thousands of cases). Thus, our final sample of 296 valid responses satisfies the methodological requirements for robust configurational analysis.

To further justify our methodological choice, we compare fsQCA with alternative approaches commonly used for causal complexity analysis, such as DEMATEL-ISM (decision making trial and evaluation laboratory and interpretive structural modeling) and DEMATEL-ANP (decision making trial and evaluation laboratory and analytic network process methods) [62]. These two methods based on DEMATEL (decision making trial and evaluation laboratory) only assume asymmetric causal relationships in a limited sense, and their main design purpose lies in factor priority ranking and structural modeling rather than revealing multiple equivalent configurations. In contrast, fsQCA is explicitly based on the logic of “joint causality” and “multiple concurrent” (reaching the same goal through different means), capable of explaining how different condition combinations lead to the same “high consultation efficiency” result. This aligns with the research objective of this study, which is to explore the collaborative dependence of multiple factors, multiple equivalent improvement paths, and the substitution relationships between conditions. It can effectively reveal the nonlinear and complex interaction mechanism behind the human-AI collaborative consultation efficiency. The mathematical formulation of the fsQCA analytical procedure is as follows:

Step 1: data calibration:


(1) $$\begin{array}{c} \mu \text{=}calibrate\left(V_{i},n_{1},n_{2},n_{3}\right) \end{array}$$

$V_{i}$ (i=1, 2, 3, … N; N denotes the number of cases) is the original variable value, $n_{1}$ is the full membership threshold, and $n_{2}$ is the intersection point. $n_{3}$ is the threshold for complete nonmembership; μ represents the fuzzy membership degree.

Step 2: necessary condition analysis:


(2) $$\begin{array}{c} Consistency_{nec}\left(X\leq Y\right)\text{=}\frac{\sum min\left(\mu_{X,}\mu_{Y}\right)}{\sum \mu_{Y}} \end{array}$$

(3) $$\begin{array}{c} Coverage_{nec}\left(X\leq Y\right)\text{=}\frac{\sum min\left(\mu_{X,}\mu_{Y}\right)}{\sum \mu_{X}} \end{array}$$

Step 3: sufficiency analysis:

A: configuration


(4) $$\begin{array}{c} Consistency_{suf}\left(A\leq Y\right)\text{=}\frac{\sum min\left(\mu_{A,}\mu_{Y}\right)}{\sum \mu_{A}} \end{array}$$

(5) $$\begin{array}{c} Coverage_{suf}\left(X\leq Y\right)\text{=}\frac{\sum min\left(\mu_{A,}\mu_{Y}\right)}{\sum \mu_{Y}} \end{array}$$

For multiple paths P1, …, Pm, overall solution consistency, solution coverage, and the unique coverage of individual paths are as follows:


(6) $$\begin{array}{c} Consistency_{sol}\text{=}\frac{\sum min\left(\mu_{A,}\mu_{Y}\right)}{\sum \mu_{A}} \end{array}$$

(7) $$\begin{array}{c} Coverage_{sol}\text{=}\frac{\sum min\left(\mu_{A,}\mu_{Y}\right)}{\sum \mu_{Y}} \end{array}$$

(8) $$\begin{array}{c} Unique\text{\textbackslash{} }Coverage_{p}\text{=}\frac{\sum min\left(\mu_{p,}\mu_{Y},1\text{-}\mu_{A\text{-}p}\right)}{\sum \mu_{Y}} \end{array}$$

Among them, ${\mu_{A\text{-}p}\text{=}max}_{q\neq p}(\mu_{q})$, measures the independent explanatory power of path p for the result Y.

Step 4: Boolean minimization output:


(9) $$\begin{array}{c} Y\text{=}P_{1}\text{+}P_{2}\text{+}\ldots \text{+}P_{m} \end{array}$$

where each $P_{i}$ represents a conjunction of several conditions.

### Questionnaire Design

Questionnaire surveys were adopted for data collection, and the questionnaire was designed based on relevant theories and empirical studies, aiming to systematically measure patients’ satisfaction with human-AI collaborative consultation services in OHCs and their influencing factors. The questionnaire consists of three parts: the first part includes screening questions to identify the target population, the second part measures patients’ satisfaction, and the third part collects demographic information. Additionally, the core variables of the questionnaire survey include 14 items in total, namely PSA, PSE, PSEF, PU, IA, IUS, IUD, LRP, PEN, SRs, SYR, SYS, OPC, and PS. A 5-point Likert scale was used in the questionnaire, where scores from 1 to 5 represent “strongly disagree” to “strongly agree,” respectively, and each variable was equipped with 3 to 4 items for measurement. To ensure the reliability and scientific quality of the questionnaire, a pretest was conducted in this study: ten college students who had used the relevant services were selected as samples to collect feedback, and after collecting feedback, three experts in the medical field were invited to revise the questionnaire, mainly adjusting items with ambiguity or problems. The revised questionnaire was then used in the subsequent large-scale survey, and the specific measurement indicators and their sources are presented in Table 2.

**Table 2.** Items and sources of the scale.

Variable	Item	Reference source
Technical factors		
SYR^a^	SYR1: the human-AI collaborative consultation system responds promptly to my operations (eg, clicks, inputs, voice commands). SYR2: the human-AI collaborative consultation system allows me to perceive “being processed” through clear prompts (eg, loading animations, progress bars, voice feedback). SYR3: during human-AI collaborative consultation, there is no obvious lag or delay when sliding or switching pages. SYR4: when asking consecutive questions, the human-AI collaborative consultation system can quickly connect to historical conversations and provide targeted responses.	Zhang et al [7], 2016; Zheng [37], 2024; Lyu [9], 2022
SYS^b^	SYS1: the human-AI collaborative consultation system runs smoothly and can stably meet my consultation needs. SYS2: the human-AI collaborative consultation system has no crashes or freezes during operation. SYS3: the system performs consistently across different devices (eg, mobile phones, tablets, computers) or browsers.	DeLone and McLean [63], 1992; Delone and McLean [58], 2003; Zhang et al [7], 2016; Hu [49], 2019
OPC^c^	OPC1: the steps required to complete a human-AI collaborative consultation are simple. OPC2: the system’s navigation design can quickly guide me to the human-AI collaborative consultation module and related auxiliary function areas. OPC3: the interface layout of the human-AI collaborative consultation module is clear, and I can use it without additional learning.	He [34], 2020; Deng and Hong [8], 2017
Information factors		
IA^d^	IA1: the human-AI collaborative consultation system describes my symptoms accurately. IA2: the human-AI collaborative consultation system provides accurate diagnosis results for my condition. IA3: the prescriptions issued by the human-AI collaborative consultation system are accurate. IA4: the suggestions provided by the human-AI collaborative consultation system are practical and actionable.	Hu [49], 2019
IUS^e^	IUS1: the information provided by the human-AI collaborative consultation system is helpful for my life and has effectively improved my daily health management. IUS2: the information provided by the human-AI collaborative consultation system has effectively alleviated my anxiety about my health status. IUS3: the health information and solutions provided by the human-AI collaborative consultation have helped me solve health problems. IUS4: the information provided by the human-AI collaborative consultation can better guide my treatment.	Jiang et al [22], 2020; Venkatesh and Bala [64], 2008; Fang et al [65], 2023
IUD^f^	IUD1: the information provided by the human-AI collaborative consultation system is expressed concisely, without excessive complex terminology. IUD2: the information provided by the human-AI collaborative consultation system has a clear logic. IUD3: the information provided by the human-AI collaborative consultation system uses forms such as charts, animations, and text-image combinations to assist understanding.	Lyu [9], 2022; Li and Wan [42], 2020
Information human factors		
PSE^g^	PSE1: the human-AI collaborative consultation system provides personalized care and solutions based on my health status, consultation history, and other information. PSE2: during the human-AI collaborative consultation, I feel a service attitude that puts my interests first. PSE3: the human-AI collaborative consultation system sincerely helps me solve problems.	Parasuraman et al [66], 1985
PSA^h^	PSA1: the effect of human-AI collaborative consultation is better than I expected. PSA2: the service provided by human-AI collaborative consultation is better than I expected. PSA3: in general, my expectations for human-AI collaborative consultation in online health communities have been fulfilled.	Bhattacherjee [67], 2001; Yang [68], 2016
PSEF^i^	PSEF1: the health questions I ask or consult through the human-AI collaborative consultation system can be answered quickly in a short time. PSEF2: human-AI collaborative consultation saves my medical time cost. PSEF3: human-AI collaborative consultation improves my medical-seeking effectiveness.	Zhang [69], 2020
PU^j^	PU1: I am worried that the human-AI collaborative consultation service may misdiagnose and give me wrong judgments. PU2: I am worried that my personal privacy may be leaked during the use of the human-AI collaborative consultation service. PU3: I am worried that using human-AI collaborative consultation will delay my best treatment time. PU4: I am worried that the online human-AI collaborative consultation service will waste money.	Ouyang and Yang [35], 2024
Environmental factors		
LRP^k^	LRP1: comprehensive online medical supervision policies have effectively protected my legitimate rights and interests during the human-AI collaborative consultation. LRP2: the standardized management of human-AI collaborative consultation and treatment data by medical policies makes me not need to worry about the risk of health information leakage. LRP3: sound laws and regulations clearly define the rights and responsibilities of doctors and patients in human-AI collaborative consultation, improving the credibility of consultation services.	He [34], 2020; Lyu [9], 2022
PEN^l^	PEN1: the human-AI collaborative consultation platform clearly publishes privacy protection policies. PEN2: the community can formulate comprehensive management regulations and punishment mechanisms to prevent internal employees, doctors, and relevant third parties from maliciously leaking user information. PEN3: in human-AI collaborative consultation, the platform collects only information directly related to diagnosis and treatment.	Zheng [37], 2024; Hu [49], 2019; Tang [70], 2021
SR^m^	SR1: public media publicity has prompted me to use the human-AI collaborative consultation service. SR2: the widespread use of human-AI collaborative consultation services by people around me affects my willingness to use it. SR3: the evaluations of human-AI collaborative consultation services by people around me affect whether I use the service.	He [34], 2020
Outcome variable		
PS^n^	PS1: I am satisfied with the decision of online health communities to adopt human-AI collaborative consultation. PS2: I think the decision of online health communities to apply human-AI collaborative consultation is wise. PS3: my experience of using human-AI collaborative consultation is pleasant. PS4: in general, I am satisfied with the application of human-AI collaborative consultation in online health communities.	Bhattacherjee [67], 2001; Yang [68], 2016

a SYR: system responsiveness.

b SYS: system stability.

c OPC: operational convenience.

d IA: information accuracy.

e IUS: information usefulness.

f IUD: information understandability.

g PSE: perceived service empathy.

h PSA: perceived service accuracy.

i PSEF: perceived service effectiveness.

j PU: perceived uncertainty.

k LRP: legal and regulatory protection.

l PEN: platform ethical norm.

m SR: social rule.

n PS: patient satisfaction.

### Recruitment

The questionnaire was administered electronically and distributed through multiple channels, including OHCs and social media groups, using Wenjuanxing (Online Questionnaire Platform). The respondents were patients who had used the human-AI collaborative consultation service. The data collection period was from May 1, 2025, to June 22, 2025, spanning 52 days. A total of 552 questionnaires were collected. After screening, responses that indicated no prior use of the relevant services, had excessively short completion times, or exhibited regular response patterns were excluded. Consequently, 296 valid questionnaires were obtained, yielding a valid response rate of 53.6% (296/552).

### Statistical Analysis

#### Reliability and Validity Analysis

Data processing and analysis were conducted using Amos (version 26.0; IBM Corp) and SPSS (version 26.0; IBM Corp). The reliability of the scale was evaluated using the Cronbach *α* coefficient and composite reliability (CR). The results indicated that the Cronbach *α* coefficient and CR values of each variable were all >0.8, indicating that the scale had good internal consistency (Table 3). Validity analysis encompassed content validity, construct validity, convergent validity, and discriminant validity. In terms of content validity, the questionnaire scale was designed based on mature scales and was optimized through translation-backtranslation procedures and a pilot study, thus demonstrating relatively high content validity. Construct validity was tested by confirmatory factor analysis (CFA), which yielded a Kaiser-Meyer-Olkin test value of 0.970 and a significant Bartlett sphericity test result (*P*<.001), indicating that the data were suitable for factor analysis (Table 4). The CFA results showed the following values: *χ*^2^_943_=1.264, root-mean-square error of approximation=0.03, goodness-of-fit index=0.86, normed fit index=0.915, comparative fit index=0.981, and Tucker-Lewis index=0.978, suggesting a good model fit (Table 5). Regarding convergent validity, the factor loadings of each item are all >0.5, the average variance extracted values were all >0.5, and the CR values were all >0.8 (Table 6). The discriminant validity test revealed that the square roots of average variance extracted for each variable were all greater than their correlations with other variables (Table 4), indicating that the scale demonstrated good discriminant validity.

**Table 3.** Reliability and validity test.

	Observed variable	Factor loading	Cronbach α	CR^a^	AVE^b^
IA^c^			0.911	0.937	0.789
	IA1	0.928			
	IA2	0.880			
	IA3	0.888			
	IA4	0.855			
IUD^d^			0.896	0.935	0.827
	IUD1	0.922			
	IUD2	0.904			
	IUD3	0.903			
IUS^e^			0.917	0.941	0.801
	IUS1	0.932			
	IUS2	0.874			
	IUS3	0.872			
	IUS4	0.901			
LRP^f^			0.917	0.948	0.858
	LRP1	0.940			
	LRP2	0.914			
	LRP3	0.924			
PEN^g^			0.884	0.928	0.812
	PEN1	0.919			
	PEN2	0.894			
	PEN3	0.890			
SR^h^			0.906	0.941	0.842
	SR1	0.923			
	SR2	0.912			
	SR3	0.917			
PSA^i^			0.905	0.905	0.840
	PSA1	0.928			
	PSA2	0.919			
	PSA3	0.902			
PSE^j^			0.883	0.928	0.811
	PSE1	0.911			
	PSE2	0.893			
	PSE3	0.897			
PSEF^k^			0.915	0.946	0.855
	PSEF1	0.936			
	PSEF2	0.923			
	PSEF3	0.914			
PU^l^			0.902	0.932	0.773
	PU1	0.925			
	PU2	0.888			
	PU3	0.876			
	PU4	0.825			
SYR^m^			0.928	0.949	0.823
	SYR1	0.935			
	SYR2	0.886			
	SYR3	0.910			
	SYR4	0.897			
SYS^n^			0.898	0.936	0.831
	SYS1	0.918			
	SYS2	0.916			
	SYS3	0.901			
OPC^o^			0.898	0.936	0.831
	OPC1	0.941			
	OPC2	0.900			
	OPC3	0.893			
PS^p^			0.916	0.941	0.798
	PS1	0.924			
	PS2	0.875			
	PS3	0.904			
	PS4	0.870			
Overall			0.982		

a CR: composite reliability.

b AVE: average variance extracted.

c IA: information accuracy.

d IUD: information understandability.

e IUS: information usefulness.

f LRP: legal and regulatory protection.

g PEN: platform ethical norm.

h SR: social rule.

i PSA: perceived service accuracy.

j PSE: perceived service empathy.

k PSEF: perceived service effectiveness.

l PU: perceived uncertainty.

m SYR: system responsiveness.

n SYS: system stability.

o OPC: operational convenience.

p PS: patient satisfaction.

**Table 4.** Correlation coefficient between the arithmetic square root of AVE^a^ and variables.

	IA^b^	IUD^c^	IUS^d^	PEN^e^	PS^f^	PU^g^	PSA^h^	PSEF^i^	PSE^j^	OPC^k^	LRP^l^	SR^m^	SYR^n^	SYS^o^
IA	0.888^p^	0.712^q^	0.645^q^	0.617^q^	0.620^q^	0.568^q^	0.641^q^	0.638^q^	0.647^q^	0.640^q^	0.600^q^	0.640^q^	0.693^q^	0.642^q^
IUD	0.712^q^	0.910^p^	0.655^q^	0.667^q^	0.712^q^	0.604^q^	0.699^q^	0.690^q^	0.672^q^	0.742^q^	0.669^q^	0.678^q^	0.702^q^	0.677^q^
IUS	0.645^q^	0.655^q^	0.895^p^	0.662^q^	0.669^q^	0.557^q^	0.667^q^	0.589^q^	0.705^q^	0.667^q^	0.665^q^	0.643^q^	0.704^q^	0.712^q^
PEN	0.617^q^	0.667^q^	0.662^q^	0.901^p^	0.699^q^	0.551^q^	0.676^q^	0.653^q^	0.703^q^	0.662^q^	0.661^q^	0.644^q^	0.678^q^	0.661^q^
PS	0.620^q^	0.712^q^	0.669^q^	0.699^q^	0.894^p^	0.571^q^	0.654^q^	0.617^q^	0.693^q^	0.710^q^	0.643^q^	0.675^q^	0.706^q^	0.684^q^
PU	0.568^q^	0.604^q^	0.557^q^	0.551^q^	0.571^q^	0.879^p^	0.547^q^	0.586^q^	0.554^q^	0.559^q^	0.488^q^	0.598^q^	0.559^q^	0.573^q^
PSA	0.641^q^	0.699^q^	0.667^q^	0.676^q^	0.654^q^	0.547^q^	0.916^p^	0.705^q^	0.728^q^	0.633^q^	0.642^q^	0.640^q^	0.707^q^	0.695^q^
PSEF	0.638^q^	0.690^q^	0.589^q^	0.653^q^	0.617^q^	0.586^q^	0.705^q^	0.925^p^	0.684^q^	0.641^q^	0.633^q^	0.611^q^	0.647^q^	0.628^q^
PSE	0.647^q^	0.672^q^	0.705^q^	0.703^q^	0.693^q^	0.554^q^	0.728^q^	0.684^q^	0.900^p^	0.673^q^	0.687^q^	0.668^q^	0.727^q^	0.662^q^
OPC	0.640^q^	0.742^q^	0.667^q^	0.662^q^	0.710^q^	0.559^q^	0.633^q^	0.641^q^	0.673^q^	0.912^p^	0.634^q^	0.678^q^	0.717^q^	0.674^q^
LRP	0.600^q^	0.669^q^	0.665^q^	0.661^q^	0.643^q^	0.488^q^	0.642^q^	0.633^q^	0.687^q^	0.634^q^	0.926^p^	0.632^q^	0.679^q^	0.684^q^
SR	0.640^q^	0.678^q^	0.643^q^	0.644^q^	0.675^q^	0.598^q^	0.640^q^	0.611^q^	0.668^q^	0.678^q^	0.632^q^	0.918^p^	0.680^q^	0.588^q^
SYR	0.693^q^	0.702^q^	0.704^q^	0.678^q^	0.706^q^	0.559^q^	0.707^q^	0.647^q^	0.727^q^	0.717^q^	0.679^q^	0.680^q^	0.907^p^	0.694^q^
SYS	0.642^q^	0.677^q^	0.712^q^	0.661^q^	0.684^q^	0.573^q^	0.695^q^	0.628^q^	0.662^q^	0.674^q^	0.684^q^	0.588^q^	0.694^q^	0.911^p^

a AVE: average variance extracted.

b IA: information accuracy.

c IUD: information understandability.

d IUS: information usefulness.

e PEN: platform ethical norm.

f PS: patient satisfaction.

g PU: perceived uncertainty.

h PSA: perceived service accuracy.

i PSEF: perceived service effectiveness.

j PSE: perceived service empathy.

k OPC: operational convenience.

l LRP: legal and regulatory protection.

m SR: social rule.

n SYR: system responsiveness.

o SYS: system stability.

p Square roots of the average variance extracted of the corresponding variables.

q *P*<.001.

**Table 5.** Common method bias test.

	Single-factor confirmatory factor analysis model	Original fitting model	Reference standard value
*χ*^2^_943_^a^/df ratio	4.045	1.264	<3
RMSEA^b^	0.102	0.03	<0.08
GFI^c^	0.595	0.86	>0.8
NFI^d^	0.702	0.915	>0.9
CFI^e^	0.757	0.981	>0.9
IFI^f^	0.758	0.981	>0.9
TLI^g^	0.746	0.978	>0.9
RFI^h^	0.688	0.903	>0.9
RMR^i^	0.086	0.038	<0.05

a Degrees of freedom are 943.

b RMSEA: root-mean-square error of approximation.

c GFI: goodness-of-fit index.

d NFI: normed fit index.

e CFI: comparative fit index.

f IFI: incremental fit index.

g TLI: Tucker-Lewis index.

h RFI: relative fit index.

i RMR: root-mean-square residual.

**Table 6.** KMO^a^ and Bartlett sphericity tests.

Item	Value
KMO measure of sampling adequacy	0.970
Bartlett test of sphericity	
Approximate chi-square (*df*)	13244.905 (1081)
Significance	0

a KMO: Kaiser-Meyer-Olkin.

#### Common Method Bias Test

As all the questionnaire data obtained were filled out by the participants within the same time period, there may be homologous bias. To verify whether there is a common method bias in the data, the single-factor CFA method was adopted. All measurement items were loaded onto a single latent factor to test the degree of model fitting. If the model fits well, it indicates that there may be significant common method bias. According to Table 7, all the fitting indicators of the original model (*χ*^2^_943_=1.264, root-mean-square error of approximation=0.03, comparative fit index=0.981, Tucker-Lewis index=0.978) are significantly better than those of the single-factor model. The fitting results of the single-factor model are poor, indicating that there is no serious common method bias problem in the data.

**Table 7.** Sample description statistics (N=296).

Options	Values, n (%)
Gender	
Male	131 (44.3)
Female	165 (55.7)
Age (years)	
Younger than 18	4 (1.4)
18‐24	86 (29.1)
25‐34	112 (37.8)
35‐50	67 (22.6)
51 and older	27 (9.1)
Education level	
High school and below	38 (12.8)
Junior college	67 (22.6)
Bachelor’s degree	132 (44.6)
Master’s degree	47 (15.9)
Doctor’s degree	12 (4.1)
Occupation	
Student	53 (17.9)
Enterprise employee	120 (40.5)
Civil servant/institution staff	45 (15.2)
Self-employed person	25 (8.4)
Others	53 (17.9)

#### Variable Calibration

To meet the Boolean logic requirements for qualitative comparative analysis, variables must be transformed into sets, and cases must be assigned to these sets before conducting fsQCA—a process known as data calibration. In this process, the values of variables must be constrained to the [0,1] interval (where 0 indicates full nonmembership and 1 indicates full membership). Given that a Likert five-point scale was adopted, the questionnaire data required calibration. First, the mean values of each measurement term are taken as variable reflection values. Considering the relationship between the calibration anchor points and the actual distribution of the samples [71], the direct calibration method is adopted, and the 0.95, 0.5, and 0.05 quantiles are respectively taken as complete membership points, intersection points, and complete nonmembership points [72]. To avoid the situation where the membership fraction is 0.5, subtract 0.001 from the calibrated value of 0.5 [73]. The entire procedure was executed with the calibrate (x, n1, n2, n3) routine in fsQCA 4.1.

### Ethical Considerations

This study was conducted in full compliance with the Declaration of Helsinki. All research protocols were reviewed and approved by the Medical Ethics Review Committee of Henan University of Science and Technology (2026-008). Informed consent was obtained from every participant. No personally identifiable information was collected, and all data were used solely for research purposes.

## Results

### User Statistics

This study collected a total of 296 valid samples, and their key characteristics were highly consistent with the core active user profile of OHCs in our country. The sample mainly consists of young and middle-aged individuals (with 198/296, 67.9%, falling within the 18‐34 years age range) and those with higher education levels (258/296, 87.2%, having a college degree or above). The sources of occupation are diverse, and the gender distribution is relatively balanced (female 165/296, 55.7%; male 131/296, 44.3%). This sample structure enables the research results to effectively reflect the current main audience group of “internet + health care,” namely, the young and middle-aged people with higher education levels, regarding their perception and evaluation of the human-AI collaborative consultation [74,75]. Therefore, the conclusions of this study are universally applicable across the country in similar digital health service scenarios. The specific characteristics of the samples are shown in Table 7.

### Necessary Condition

#### Overview

In accordance with the fsQCA method, before conducting the configurational analysis, the first step is to perform a necessity analysis of the individual condition variables, with the results expressed through consistency and coverage. Consistency represents the degree to which the condition variables are a subset of the outcome variables. Identifying a necessary condition generally requires a consistency score higher than 0.9 [76]. Coverage represents the extent to which the condition variables explain the outcome. Coverage is meaningful only for conditions that satisfy the consistency requirement, and no generally accepted threshold exists.

Necessity analysis of the calibrated data was performed using fsQCA 4.1 software. The analysis results show that the consistency scores of all condition variables have not reached the necessary standard of 0.9, indicating that no single condition variable independently constitutes a necessary condition for PS. Therefore, further exploration of how combinations of condition variables affect PS is required through configurational path analysis. Table 8 presents the results of the necessity test for individual condition variables.

**Table 8.** Results of single-factor necessity analysis.

Conditional variable	High patient satisfaction	
	Consistency	Coverage
SRfs^a^	0.819797	0.805465
~^b^SRfs	0.526479	0.527170
~PENfs^c^	0.590319	0.546169
PENfs	0.781640	0.835399
~LRPAfs^d^	0.512780	0.537554
LRPAfs	0.826889	0.778196
~PUfs^e^	0.559792	0.566387
PUfs	0.781238	0.759865
~PSEFfs^f^	0.484637	0.513980
PSEFfs	0.829484	0.772638
~PSAfs^g^	0.528298	0.537494
PSAfs	0.814435	0.787966
~PSEfs^h^	0.486123	0.508451
PSEfs	0.850344	0.801910
~IUDfs^i^	0.507990	0.521243
IUDfs	0.836992	0.803325
~IUSfs^j^	0.530199	0.525761
IUSfs	0.816077	0.809565
~IAfs^k^	0.534396	0.535304
IAfs	0.813312	0.798788
~OPCfs^l^	0.575359	0.535996
OPCfs	0.788834	0.836473
~SYSfs^m^	0.502152	0.521759
SYSfs	0.833361	0.790617
~SYRfs^n^	0.493855	0.515469
SYRfs	0.841999	0.795528

a SRfs: high social rule.

b ~: indicates the negation of the condition.

c PENfs: high platform ethical norm.

d LRPAfs: high legal and regulatory protection.

e PUfs: high perceived uncertainty.

f PSEFfs: high perceived service effectiveness.

g PSAfs: high perceived service accuracy.

h PSEfs: high perceived service empathy.

i IUDfs: high information understandability.

j IUSfs: high information usefulness.

k IAfs: high information accuracy.

l OPCfs: high operational convenience.

m SYSfs: high system stability.

n SYRfs: high system responsiveness.

#### Adequacy Analysis of Configuration

After completing the univariate necessity analysis, the fsQCA 4.1 software was further used for configuration analysis to explore the sufficiency effect of variable combinations on PS. Referring to the configuration analysis method of Ragin [71], the consistency threshold was set to 0.8, the proportional reduction in inconsistency value to 0.7, and the case threshold to 2. A truth table was constructed, and the configuration solution was generated. The analysis results show that the consistency of all five configurations is higher than 0.9, and the overall consistency is 0.962, significantly higher than the threshold value of 0.75, indicating that these configurations constitute sufficient conditions. Meanwhile, the overall coverage rate was 0.544, indicating that the research results could explain more than half of the cases in the sample. By distinguishing the core conditions that occur simultaneously in the intermediate solution and the parsimony solution from the auxiliary conditions that only appear in the intermediate solution, the configurations with the same core conditions are classified, and ultimately, five types of antecedent configuration patterns that trigger high PS are obtained (Table 9).

**Table 9.** Configuration paths for high patient satisfaction.

Antecedent conditions	High patient satisfaction^a^				
	Configuration 1	Configuration 2	Configuration 3	Configuration 4	Configuration 5
Technical factors					
SYR^b^	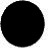 ^c^	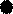	●^d^	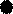	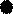
SYS^e^	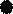	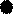	●	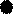	⊗^f^
OPC^g^	—^h^	⊗	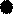	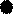	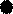
Information factors					
IA^i^	●	●	●	●	●
IUS^j^	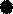	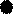	●	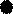	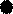
IUD^k^	●	●	●	●	●
Information human factors					
PSE^l^	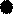	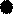	●	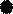	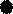
PSA^m^	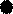	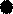	●	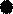	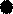
PSEF^n^	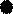	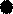	●	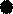	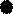
PU^o^	●	—	●	—	●
Environmental factors					
LRP^p^	●	●	●	●	●
PEN^q^	—	⊗	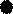	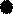	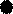
SR^r^	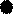	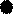	—	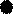	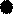
Raw coverage of configuration	0.480	0.290	0.464	0.490	0.256
Unique coverage of configuration	0.015	0.000	0.014	0.031	0.009
Consistency	0.964	0.979	0.977	0.982	0.989

a Solution coverage=0.544 and solution consistency=0.962.

b SYR: system responsiveness.

c Denotes the presence of a core antecedent condition.

d Denotes the presence of a peripheral antecedent condition.

e SYS: system stability.

f Denotes the absence of a peripheral antecedent condition.

g OPC: operational convenience.

h Indicates that whether the condition is present or absent has no impact on the outcome variable.

i IA: information accuracy.

j IUS: information usefulness.

k IUD: information understandability.

l PSE: perceived service empathy.

m PSA: perceived service accuracy.

n PSEF: perceived service effectiveness.

o PU: perceived uncertainty.

p LRP: legal and regulatory protection.

q PEN: platform ethical norm.

r SR: social rule.

#### Comparison Among Configurations for High PS

From a single-factor perspective, in the configuration leading to high PS, the following conditions are all core or important: high SYR, high IUS, high PSE, high PSA, and high PSEF. High IA, high IUD, and high LRP are auxiliary conditions. It is worth noting that neither nonhigh PU nor nonhigh SRs appear in the configuration. From the perspective of factor combination, a comparison between configurations 3 and 4 reveals that, given the simultaneous presence of SYR, SYS, OPC, IUS, IA, IUD, PSE, PSA, PSEF, LRP, and PEN, PU and SRs exert a substitution effect in enhancing PS.

#### Configuration Analysis of High PS

Based on configuration characteristics and driving logic, five realization paths for high PS were identified, and the generation mechanisms of PS were revealed from the dimensions of technology, information, service, and environment, respectively.

Regarding the technology-information human-driven type guided by SRs (configuration 1), the core antecedent conditions of configuration 1 include SYR, SYS, PSE, PSA, PSEF, and SRs. SYR and stability provide patients with a favorable technical experience, ensuring that they receive rapid feedback and a continuous and stable service experience during use. This effectively avoids negative emotions caused by lag, delay, or system crash and enhances overall usability and trust. On this basis, the empathy, accuracy, and effectiveness of perceived services, as key manifestations of the “serving people” dimension, address patients’ multidimensional needs in terms of emotional resonance, professional judgment, and response speed. As an external environmental factor, social norms play a guiding and reinforcing role in this configuration. Through social transmission mechanisms such as recommendations from relatives and friends, public evaluation, and media coverage, a positive cognitive atmosphere regarding the human-AI collaborative consultation model is created, which enhances patients’ willingness to adopt and confidence in using the system. Although IA, IUD, and LRP are marginal conditions, they provide patients with a reliable and orderly medical environment through their basic supporting role. Furthermore, the impact of PU in this configuration is relatively weak, indicating that the effective combination of the above core conditions has successfully alleviated patients’ concerns about service uncertainty, achieving a transition from “technology availability” to “service trustworthiness.” This fully demonstrates the sustainable development logic of “technology as the foundation, service as the core, and social empowerment” in human-AI collaborative consultation.

Regarding the technology-information human-driven under the inclusiveness of platform ethics (configuration 2), the core conditions of configuration 2 are identical to those of configuration 1, namely SYR, SYS, PSE, PSA, PSEF, and SRs, indicating that technical reliability and service quality perception play universal and key roles in human-AI collaborative consultation. The difference is that in configuration 2, OPC and PEN are not core conditions; in some cases, they are even absent, reflecting that there exists a “compensation mechanism” and “priority differentiation” in achieving PS. Our findings show that although platform operation is somewhat complex, the system’s rapid response and stability can effectively offset the negative impact of the operational threshold, embodying the compensation logic that “efficient response is a form of user experience optimization.” Furthermore, the absence of marginal ethical norms on the platform indicates that, in specific usage scenarios, patients tend to prioritize immediate and tangible service value over abstract long-term ethical guarantees. This phenomenon is particularly evident among user groups that primarily receive low-risk, high-frequency services such as daily health consultations, minor illness diagnoses, and medication advice. This finding indicates that, in specific user demand scenarios, a good service experience itself can partially substitute for the construction of institutional trust, forming a trust generation mechanism whereby “service temperature offsets the cost of norms.” This path highlights the need for smart medical services to be tailored to specific needs. For different user groups and usage scenarios, while maintaining the basic compliance bottom line, priority should be given to strengthening technical performance and service quality to achieve a dynamic balance between effectiveness and sustainability.

Regarding the technology-environment driven type under the guidance of platform specifications (configuration 3), configuration 3 reveals a satisfaction realization path driven by “operational convenience” and “platform ethical norms,” “platform ethical norms,” highlighting the fundamental role of technology accessibility and system credibility in shaping PS among specific user groups and usage scenarios. The convenience of operation plays a key role as “entry empowerment” in this path. By simplifying the consultation process, it enables older adult patients, individuals with low digital literacy, or those seeking help for acute conditions—who may be less familiar with the consultation system—to easily initiate consultations, upload materials, and so on, thereby significantly reducing the usage barriers. This allows them to initiate consultations, submit health information, and receive feedback smoothly, thus enhancing the accessibility and inclusiveness of services. PEN serves as the core support at the environmental level, providing institutional guarantees regarding data privacy protection, algorithmic fairness, service transparency, and accountability traceability. Although they do not directly participate in the service interaction process, they establish a “trusted foundation” for the entire consultation ecosystem. Furthermore, information factors and service perception factors are in a marginal position in this configuration, indicating that when basic availability and institutional security are not yet fully satisfied, higher-level service experience is unlikely to become the main factor influencing satisfaction. This model is particularly suitable for scenarios such as community medical mini-programs and grassroots health service platforms that offer low-frequency and lightweight services to the general public. Its success lies in winning users’ trust through “simplicity + reliability.” Therefore, this path emphasizes that during the process of expanding smart health care toward inclusiveness and grassroots levels, the platform should prioritize building a “low-threshold and highly trustworthy” infrastructure, taking OPC and ethical norms as the key levers to enhance the satisfaction of vulnerable groups, thereby providing strong support for achieving health equity and sustainable development.

Regarding the all-element collaborative drive type under demand-oriented logic (configuration 4), configuration 4 embodies a “full-chain, high-standard” service logic for high-value and high-stickiness users, and represents the highest-level pathway to achieving the effectiveness of human-AI collaborative consultation. Its core feature lies in the comprehensive coordination and deep integration of the four dimensions—technology, information, service, and environment—forming a closed-loop service system characterized by “stable technology, solid information, warm humanistic atmosphere, and excellent environment.” SYR, stability, and OPC build a technical experience advantage. IUS meets patients’ core health demands. Perceiving service elements strengthens the humanistic care of services. PEN and SRs further optimize the service environment. This path is particularly suitable for in-depth health service scenarios such as chronic disease management, long-term follow-up, and rehabilitation guidance. Its users often have a strong dependence on the platform and possess a high level of health literacy and service expectations. They are concerned not only with the immediate outcomes of a single consultation but also place great importance on service continuity, professional consistency, and trust accumulation. Consequently, any shortcoming in any link may lead to a significant decline in overall satisfaction. Configuration 4 indicates that to meet the comprehensive needs of such users, the platform must abandon the “single-point optimization” mindset and shift toward systematic and refined service design. It should provide comprehensive protection across the entire chain, take deep alignment with user needs as its orientation, and build a sustainable, high-quality service ecosystem, thereby enhancing user loyalty and the platform’s core competitiveness.

Regarding the all-element collaborative driven type made inclusive by technology stability (configuration 5), configuration 5 reveals a specific path that can still achieve high satisfaction through the synergy of other core elements against a background of relatively weakened SYS, reflecting users’ differentiated perception of service priorities at specific development stages. Its core conditions include SYR, OPC, IUS, PSE, PSA, and PSEF, forming an experience advantage centered on “quick response, easy and efficient use, trustworthy content, and empathetic service.” SYS is not a necessary condition in this configuration, indicating that during the early stage of rapid platform expansion or technological iteration, some patients have a relatively high tolerance for occasional system failures or brief service interruptions. Their satisfaction depends more on whether the platform can provide timely, accurate, and empathetic responses at critical points. This phenomenon is particularly evident among young users, individuals with acute mild symptoms, or those seeking information-based consultations. They are more concerned with the effectiveness and quality of problem-solving rather than the absolute stability of the system. Furthermore, although legal and regulatory guarantees and PEN have not emerged as core conditions, they have indirectly enhanced the sense of security in use by reducing patients’ PU, playing an “implicit support” role. It is worth noting that this path has phased characteristics and is suitable for the platform growth stage or market acquisition stage. In the long term, if SYS issues occur frequently, it will significantly weaken patients’ trust in the platform. Therefore, the platform still needs to increase technological investment, address the stability deficiencies, and achieve a transition from “function-driven” to “reliably driven” operations to ensure the sustainable improvement of service effectiveness.

### Robustness Test

To verify the robustness of the research results, a systematic test was conducted on the traction configuration of PS by adjusting the thresholds of key parameters. First, the original consistency threshold was gradually increased from 0.8 to 0.85 and 0.9, or the proportional reduction in inconsistency value was adjusted from 0.7 to 0.75 and 0.8. The results showed that the adjusted configuration analysis results remained stable, and the core conclusion did not undergo substantial changes. Second, the case frequency threshold was raised from 2 to 3. The test results showed that the three configurations generated were basically consistent with configurations 1, 2, and 4 in the original configuration, with only marginal differences in the consistency and coverage of the solutions. This verifies the robustness of the research results.

## Discussion

### Principal Results

Based on the perspective of information ecology and combined with the fsQCA method, this study systematically explored the multidimensional influence mechanism and configuration path of the human-AI collaborative consultation effectiveness in OHCs. The principal findings are as follows. First, no single antecedent condition constitutes a necessary condition for high consultation effectiveness; its achievement results from the synergistic configurations of multiple factors, confirming the principle of causal complexity and equifinality. In practice, this explains why platforms that focus on different features can all achieve high PS. Second, five distinct configuration paths leading to high consultation effectiveness were identified, which can be categorized into technology-information human-driven type guided by SRs, technology-information human-driven type made inclusive by platform ethics, technology-environment driven type guided by platform norms, all-element collaborative driven type under demand-oriented logic, and all-element collaborative driven type made inclusive by technology stability. These paths indicate that the same outcome can be achieved through different starting points, providing the platform with flexible strategic options. Third, among the various antecedent conditions, SYR, IUS, PSE, PSA, and PSEF serve as core or important driving conditions. These factors consistently emerged in multiple successful configurations, indicating that they are the core guarantees for high performance. Fourth, IA, IUD, and LRP are the common auxiliary conditions for each group. These factors alone cannot trigger success, but they provide the necessary foundational support. The results show that after meeting the basic standards, further strengthening these factors has a limited marginal improvement on efficiency. The platform should shift more resources to other differentiated conditions. Fifth, there is a substitution effect among the antecedent conditions for achieving high PS. The perceptual uncertainty between configurations 3 and 4 and SRs has a substitution effect in improving PS. The OPC of configurations 1 and 4, as well as the platform’s ethical norms and PUs, enhance PS in an equivalent substitution manner. This discovery directly addresses the issue of resource constraints in reality, indicating that platforms with limited resources can make up for their shortcomings by strengthening alternative factors.

### Practical Implications

The findings have significant implications for OHC operators and public health policymakers. First, for platform developers and technology providers, priority should be given to ensuring core system performance, particularly response speed and the usefulness of information output. This study confirms that even when other conditions (such as OPC or PEN) are relatively lacking, an efficient and useful system remains a key cornerstone driving satisfaction. Therefore, resources should be prioritized for algorithm optimization, speed improvement, and quality control of information content, to ensure that the AI system can provide health information and auxiliary diagnosis suggestions that meet the needs of patients quickly and accurately, and solve the actual problems of “slow response and mixed information” in online consultations. Second, platform operators and managers need to focus on building the “warmth” of the service experience. The perception service’s empathy, accuracy, and efficiency are the core soft power for enhancing user loyalty. This requires the platform to incorporate humanistic elements in the AI interaction design, ensuring that the responses from doctors and AI align with the emotional needs of patients. At the same time, it is necessary to strictly control the accuracy of medical advice and the effectiveness of problem-solving, breaking the “instrumentalization” barrier in human-computer interaction, and building an emotional connection and doctor-patient trust beyond the technical level. Third, platforms should adopt differentiated and context-aware service strategies. This study reveals that for users seeking convenient, efficient, and lightweight consultations, enhancing OPC and rapid response may impact satisfaction more directly than providing comprehensive ethical clauses. In contrast, for users engaged in in-depth services such as chronic disease management, high-standard coordination across all elements—technology, information, service, and environment—is required. Therefore, the platform can dynamically adjust resource allocation and functional priorities based on user profiles and service scenarios, achieving “precise adaptation and on-demand service.” Fourth, for regulators, there is an urgent need to accelerate the development of legal and ethical standards for human-AI collaborative consultation, clarify liability boundaries, and encourage platforms to establish transparent data usage and algorithmic explanation mechanisms to build public trust. Meanwhile, research has shown that SRs are a cost-effective lever for enhancing user acceptance. Platforms can encourage users to share positive consultation experiences and create a positive community interaction atmosphere, thereby forming a virtuous cycle recognized by society and further promoting the popularization of the human-AI collaborative consultation model.

### Limitations

This study has several limitations. First, the sample was drawn primarily from OHCs in China; the unique cultural and health care system context may limit the generalizability of the findings, warranting cross-cultural comparative studies in the future. Second, the cross-sectional survey design cannot capture the dynamic evolution of user satisfaction; longitudinal tracking or mixed methods (eg, interviews combined with log analysis) are recommended for deeper mechanistic exploration. Third, this study focuses solely on the patient perspective, excluding input from physicians or platform operators; future research could adopt a multistakeholder collaborative framework. Fourth, while fsQCA excels at revealing configurational effects, it cannot quantify the marginal contribution of individual conditions; integration with structural equation modeling could provide complementary validation. Fifth, this study focused on PS as the core indicator of consultation effectiveness. Future research could incorporate richer outcome variables, such as improvements in adherence, health outcome indicators, or changes in physicians’ workload, to more comprehensively assess the integrated value of human-AI collaboration.

### Comparison With Prior Work

The contributions of this study are manifested in three aspects. First was at the theoretical reconstruction level. Although the constituent variables involved in this study are rooted in a mature theoretical framework, the core innovation lies in incorporating the existing constructs into the 4D framework of the information ecology theory: “information human, information, technology, and environment,” reconceptualizing them in the context of human-AI collaboration as interdependent ecological elements, providing a holistic theoretical lens for understanding human-AI collaboration. Second was at the methodological paradigm level. Unlike traditional technology adoption research that relies on structural equation models to identify independent net effects, this study introduces fsQCA into the context of human-AI collaborative consultation, empirically revealing multiple concurrent configuration effects and causal complexity, achieving a paradigm shift from “net effect analysis” to “configuration mechanism analysis.” Third was at the context-specific level. Different from general technology adoption or traditional telemedicine, this study focuses on the unique application scenario of human-AI collaborative consultation. In this context, AI (technology) is not merely a tool; rather, it forms a deeply coupled dynamic interaction relationship with humans (patients), health information, and the platform environment. The paths of variable effects and interaction logic all align with the core requirements of health information flow, and there are fundamental differences from traditional technology applications. This discovery clarifies the boundaries between human-AI collaborative consultation and traditional technology application scenarios, providing precise contextual guidance for the optimization of digital health platforms.

### Conclusions

From the systemic perspective of information ecology and using the fsQCA method, this study has provided an in-depth analysis of the multiple driving pathways to the effectiveness of human-AI collaborative consultation in OHCs. The findings show that high consultation effectiveness results from the synergistic interaction of factors across four dimensions: technology, information, information human, and environment. Furthermore, multiple equifinal configurational paths exist, with core conditions (such as SYR, IUS, and perceived service quality) playing key roles, and important substitution relationships exist among antecedent conditions. These findings not only deepen the theoretical understanding of the mechanisms underlying digital health service effectiveness but also provide empirical evidence and actionable guidelines for OHC platforms seeking to optimize the design of human-AI collaborative services and implement differentiated operational strategies. Looking ahead, as AI technology continues to evolve and achieve deeper integration with health services, continued exploration of the dynamic interactions among multiple factors, cross-cultural differences, and long-term health impacts will further promote the development of human-AI collaboration models toward increased intelligence, human-centeredness, and sustainability, ultimately supporting the optimization of medical resources and the improvement of population health.

## Abbreviations

CFA: confirmatory factor analysis
CR: composite reliability
DEMATEL: decision making trial and evaluation laboratory
DEMATEL-ANP: decision making trial and evaluation laboratory and analytic network process methods
DEMATEL-ISM: decision making trial and evaluation laboratory and interpretive structural modeling
fsQCA: fuzzy set qualitative comparative analysis
IA: information accuracy
IUD: information understandability
IUS: information usefulness
LRP: legal and regulatory protection
OHC: online health community
OPC: operational convenience
PEN: platform ethical norm
PS: patient satisfaction
PSA: perceived service accuracy
PSE: perceived service empathy
PSEF: perceived service effectiveness
PU: perceived uncertainty
SERVQUAL: service quality theory
SR: social rule
SYR: system responsiveness
SYS: system stability
